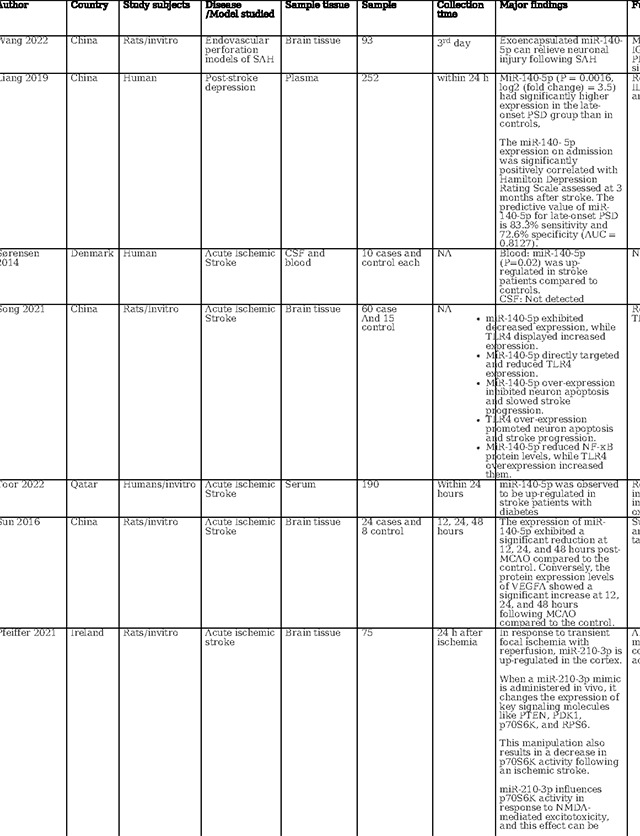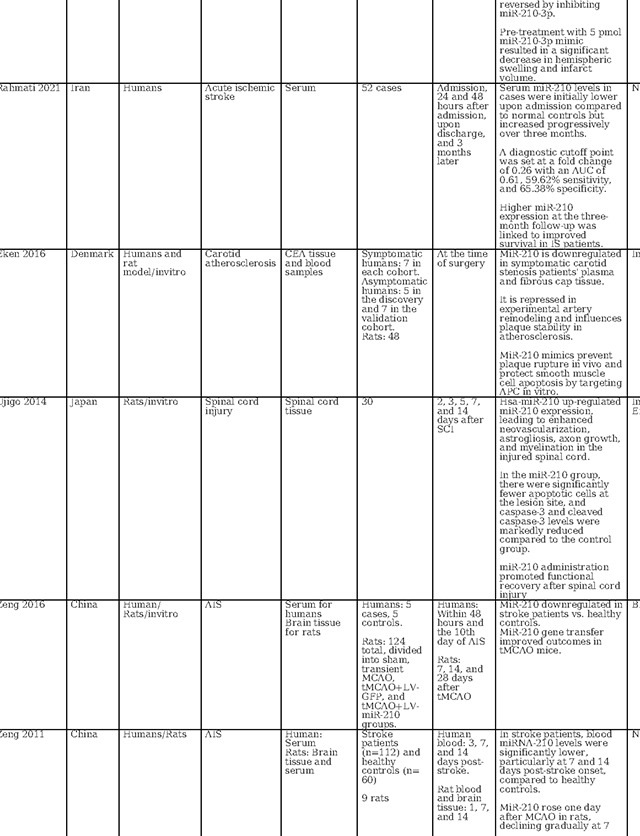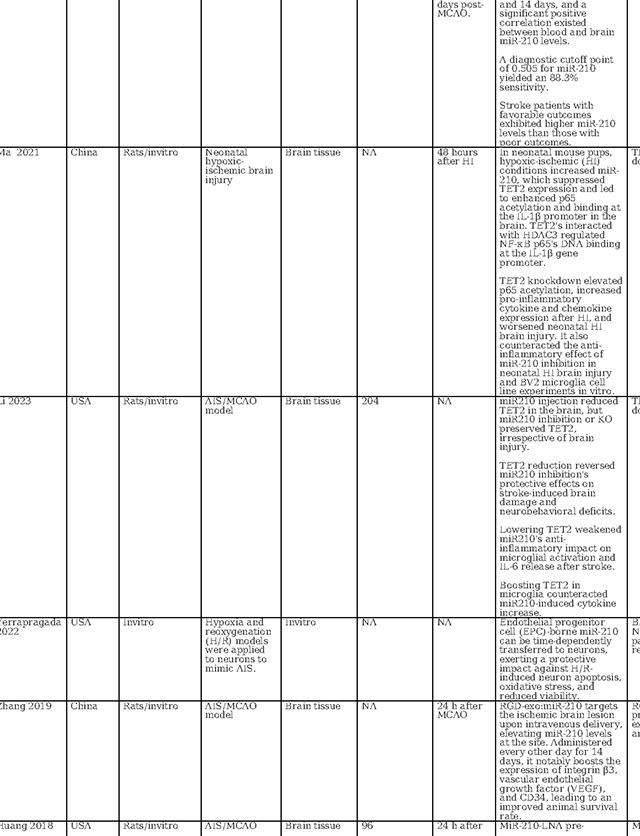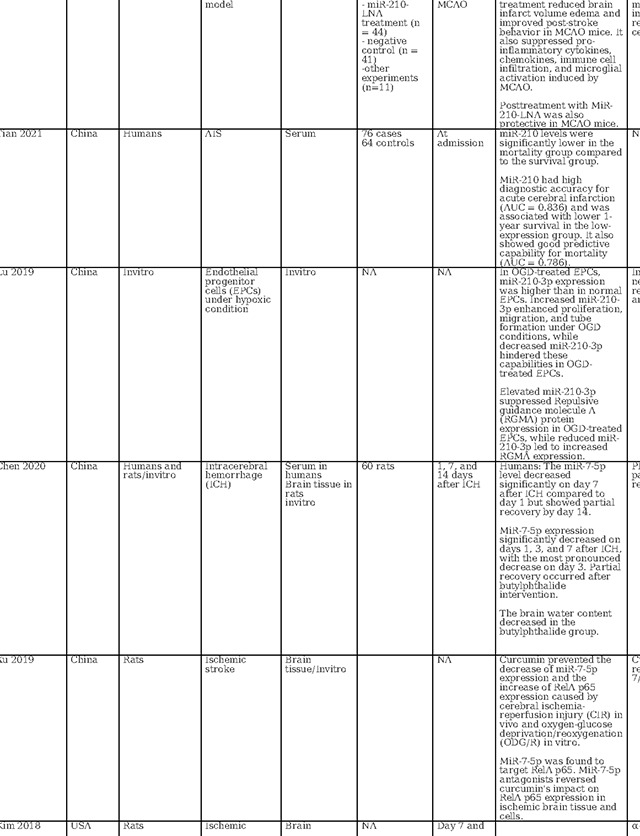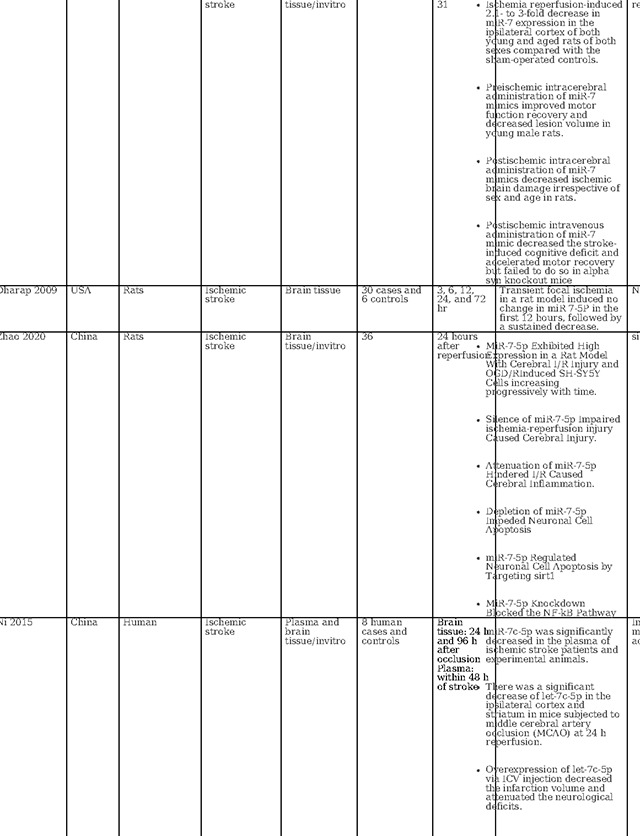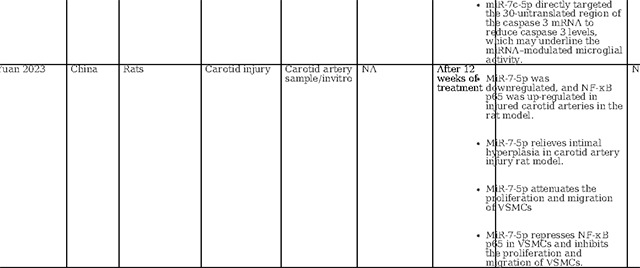# MicroRNA Expression Profile in Acute Ischemic Stroke

**DOI:** 10.21203/rs.3.rs-3754883/v1

**Published:** 2024-01-03

**Authors:** Shraddha Mainali, Gaurav Nepal, Amy Webb, Paolo Fadda, Darya Mirebrahimi, Patrick Nana-Sinkam, Brad Worrall, Daniel Woo, Nicholas Johnson, Mohammad Hamed

**Affiliations:** Virginia Commonwealth University; Tribhuvan University; The Ohio State University; The Ohio State University; Virginia Commonwealth University; Virginia Commonwealth University; University of Virginia; University of Cincinnati College of Medicine; Virginia Commonwealth University; The Ohio State University

**Keywords:** acute ischemic stroke, miRNA, biomarker, micro-RNA, miR, large vessel occlusion, LVO, stroke biomarker

## Abstract

**Introduction:**

Acute ischemic stroke with large vessel occlusion (LVO) continues to present a considerable challenge to global health, marked by substantial morbidity and mortality rates. Although definitive diagnostic markers exist in the form of neuroimaging, their expense, limited availability, and potential for diagnostic delay can often result in missed opportunities for life-saving interventions. Despite several past attempts, research efforts to date have been fraught with challenges likely due to multiple factors such as inclusion of diverse stroke types, variable onset intervals, differing pathobiologies, and a range of infarct sizes, all contributing to inconsistent circulating biomarker levels. In this context, microRNAs (miRNAs) have emerged as a promising biomarker, demonstrating potential as biomarkers across various diseases, including cancer, cardiovascular conditions, and neurological disorders. These circulating miRNAs embody a wide spectrum of pathophysiological processes, encompassing cell death, inflammation, angiogenesis, neuroprotection, brain plasticity, and blood-brain barrier integrity. This pilot study explores the utility of circulating exosome-enriched extracellular vesicle (EV) miRNAs as potential biomarkers for anterior circulation LVO (acLVO) stroke.

**Methods:**

In our longitudinal prospective cohort study, we collected data from acute large vessel occlusion (acLVO) stroke patients at four critical time intervals post-symptom onset: 0–6 hours, 6–12 hours, 12–24 hours, and 5–7 days. For comparative analysis, healthy individuals were included as control subjects. In this study, extracellular vesicles (EVs) were isolated from the plasma of participants, and the miRNAs within these EVs were profiled utilizing the NanoString nCounter system. Complementing this, a scoping review was conducted to examine the roles of specific miRNAs such as miR-140–5p, miR-210–3p, and miR-7–5p in acute ischemic stroke (AIS). This review involved a targeted PubMed search to assess their influence on crucial pathophysiological pathways in AIS, and their potential applications in diagnosis, treatment, and prognosis. The review also included an assessment of additional miRNAs linked to stroke.

**Results:**

Within the first 6 hours of symptom onset, three specific miRNAs (miR-7–5p, miR-140–5p, and miR-210–3p) exhibited significant differential expression compared to other time points and healthy controls. These miRNAs have previously been associated with neuroprotection, cellular stress responses, and tissue damage, suggesting their potential as early markers of acute ischemic stroke.

**Conclusion:**

This study highlights the potential of circulating miRNAs as blood-based biomarkers for hyperacute acLVO ischemic stroke. However, further validation in a larger, risk-matched cohort is required. Additionally, investigations are needed to assess the prognostic relevance of these miRNAs by linking their expression profiles with radiological and functional outcomes.

## Introduction

Acute ischemic stroke (AIS) is a medical emergency characterized by the sudden blockage of blood flow to the brain, resulting in high morbidity and mortality rates worldwide(1). Emergent treatment decisions are time-critical, necessitating precise determination of stroke onset time or its practical surrogate, the ‘last known well’ (LKW) time, to expedite the initiation of appropriate interventions(2). Over 30% of AIS cases involve large vessel occlusion (LVO), particularly in major arteries such as the internal carotid artery (ICA) and the anterior (ACA), middle (MCA), and posterior cerebral arteries, significantly contributing to the burden of stroke due to the large area of ischemic tissue and infarction(3).

According to current clinical guidelines, patients presenting with AIS within 4.5 hours from symptom onset are candidates for intravenous (IV) thrombolysis. Initiating IV thrombolysis within this timeframe increases the likelihood of improved functional outcomes across all age groups, with the magnitude of benefit being highly time-dependent (4,5). For patients with anterior circulation large vessel occlusion (acLVO), endovascular thrombectomy (ET) is typically indicated within 6 hours of onset without the need for advanced perfusion imaging. Beyond this window, up to 24 hours, ET may be considered based on a thorough risk/benefit assessment (4). Contemporary stent retriever devices can achieve successful recanalization in over 87% of patients, significantly enhancing outcomes with a number needed to treat (NNT) of 8 for an excellent clinical outcome and an NNT of 3 for a favorable functional outcome, without markedly increasing mortality or hemorrhagic complications(6). It is estimated that increasing the rate of near-complete to complete reperfusion by just 10% could result in an additional 3656 quality-adjusted life years (QALYs) and save $21.0 million and $36.8 million for the US healthcare system and society, respectively (7). Without timely intervention, the progression of stroke leads to rapid loss of neural tissue. In LVO patients, an estimated 120 million neurons, 830 billion synapses, and 714 km (447 miles) of myelinated fibers are lost every hour(8). As the stroke advances, the risk of intracranial hemorrhage (ICH) begins to outweigh the benefits of recanalization therapy, typically beyond 24 hours (9,10). Therefore, determination of stroke onset time is paramount in delivering safe and effective treatment for stroke patients.

Nevertheless, a substantial proportion of AIS patients, approximately one in four, present with unclear stroke onset time or LKW, making them ineligible for potentially life-saving acute stroke intervention(11). The lack of reliable methods to estimate the time of stroke onset and accurately gauge the extent of tissue injury poses a significant challenge in managing AIS effectively, especially in cases where the precise timing of symptom onset remains uncertain.

Recent literature has highlighted the significance of molecular intercellular messaging and signaling in determining the state of tissue injury in various diseases, including stroke(12). MicroRNAs (miRNAs) have emerged as a class of non-coding RNA molecules that play a pivotal role in intercellular communication by regulating the expression of target mRNAs(13). In the context of AIS, miRNAs have shown promise as potential biomarkers for diagnostic and prognostic applications(14–17). Studies have reported altered miRNA expression profiles in blood and brain tissues of AIS patients, suggesting their potential as biomarkers for stroke detection, subtyping, and prognosis (18,19). However, to date, no study has comprehensively evaluated the temporal miRNA expression profiles in hyperacute AIS patients with acLVO stroke over 7 days following symptom onset. Understanding the dynamic changes in miRNA expression during the hyperacute phase of acLVO would be highly beneficial for unraveling the early molecular signals underpinning this devastating condition. This knowledge opens avenues for potential blood-based biomarkers that could transform early diagnosis and monitor treatment efficacy. Moreover, it sheds light on the molecular underpinnings of stroke progression and tissue damage, offering opportunities for improved clinical decision-making, prognostication, and the discovery of new therapeutic targets (20). This pilot study provides critical insights into the dynamic shifts in miRNA expression patterns related to hyperacute acLVO stroke, observed longitudinally over a week.

## Methods

### Study Design

This longitudinal, prospective cohort study, conducted at the Joint Commission-certified Comprehensive Stroke Center of Ohio State University—a tertiary referral medical center—explores the viability of circulating EV-encapsulated miRNAs as blood-based biomarkers for acLVO ischemic stroke. The study protocol received approval from the Ohio State University Institutional Review Board, and informed consent was secured from all participants or their legally authorized representatives.

Our scoping review was designed to elucidate the roles of certain miRNAs, specifically miR-140–5p, miR-210–3p, and miR-7–5p, within the acute ischemic stroke (AIS) framework. To achieve this, we conducted a comprehensive PubMed search using terms like ‘acute ischemic stroke’, ‘stroke’, ‘miR’, ‘miRNA’, ‘micro RNA’, and the specific miRNAs of interest (miR-140–5p, miR-210–3p, and miR-7–5p). We employed Boolean operators “AND” and “OR” to refine the search. Our primary objective was to explore the current research on how these miRNAs influence key pathways in ischemic stroke pathophysiology, including apoptosis, inflammation, oxidative stress, and neuronal damage, and their potential roles in diagnosis, treatment, and prognosis. The results section of this manuscript presents an in-depth review of the three principal miRNAs, based on our lab research. Furthermore, we provide a summary of important findings from other stroke related miRNA in Supplementary File 1.

### Sampling and Enrollment:

The study enrolled all patients who arrived at the adult Emergency Department within 6 hours of witnessed stroke symptom onset. Inclusion required a confirmed diagnosis of acLVO by CT Angiogram. Healthy individuals formed the control group. Exclusion criteria included individuals with significant atherothrombotic disorders—such as pronounced coronary or peripheral vascular disease, deep vein thrombosis, or pulmonary embolism—patients with concurrent neurological conditions, a stroke in the preceding three months, posterior circulation LVO, uncertain time since last seen well, and pregnancy. Patients with recent thrombosis or severe atherosclerosis were excluded to avoid interference from clot associated miRNAs, like platelet-derived miRNAs. Individuals with recent brain injuries were excluded to prevent confounding results from miRNA profiles linked to these recent injuries. Pregnant women were excluded due to potential variations in miRNA profiles in expectant mothers with growing fetus.

### Sample collection and processing:

Blood samples were collected from acLVO patients at four time points: 0–6 hours, 6–12 hours, 12–24 hours, and 5–7 days after symptom onset. Controls were healthy volunteers without any acute disease process or chronic stroke risk factors and had blood samples collected at a single time point. After initial sample collection and centrifugation, plasma was stored at −80°C until further processing. Subsequently, extracellular vesicles (EV) enriched in exosomes were isolated from the cell-free plasma using the Total Exosome Isolation Kit (Invitrogen) as previously described(21). The isolated EVs were characterized for quantity and size using the NanoSight NS300 (22). Total RNA were isolated using the Maxwell RSC miRNA tissue kit (Promega)(23). MiRNA profiling was performed using the multiplexed NanoString nCounter miRNA system as previously described (22). Nanostring counts exported from nSolver were filtered and normalized with in-house scripts where miRNA and samples were filtered based on negative background probes (NegCutoffS1 = meanNegS1 + 1.5*stdevNegS1) and normalized based on the geometric mean of expression and log2 transformed.

### Statistical Analysis:

Statistics was performed in R. Heatmaps with hierarchical clustering, and principal component analysis (PCA) was performed with pheatmap and prcomp to visually group samples into clusters to give us an idea of the differences and similarities between samples and sample categories. Modest changes (< 2 fold) in miRNA expression are known to be associated with changes in target gene expression(24–26). Change of miRNAs across time points in LVO patients was assessed using repeated measures ANOVA, and we tested time point differences between any two-time points with a mixed model with a sample included as a random effect. The significance for any statistical test was defined as FDR < 0.05.

## Results

Analysis was performed on a total of 24 samples from six patients with confirmed acLVO stroke, with 12 samples from patients presenting with right ICA/MCA involvement and the remaining 12 samples with left ICA/MCA involvement. Individual patient characteristics are presented in Table 1. All patients received IV thrombolytic therapy (tPA) within the appropriate therapeutic window. Furthermore, some patients underwent endovascular thrombectomy as an additional intervention to restore blood flow, resulting in the collection of plasma samples during (1 patient) and after the thrombectomy procedure (2 patients). Control included 5 healthy volunteers (3 males, 2 females) between the ages of 18–60 years.

**Figure 1:**
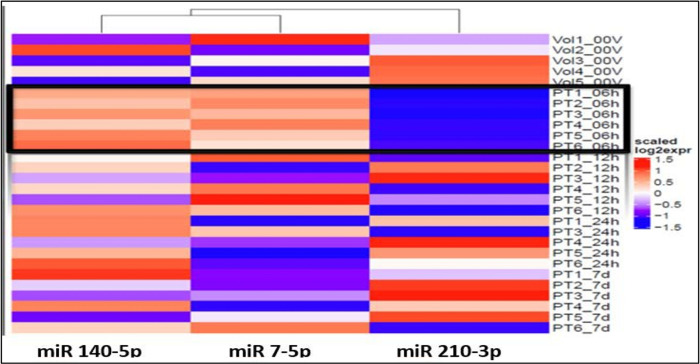
Heat map shows over-expression of miRs 140–5p and 7–5p (red) and under-expression of miR 210–3p (blue) within 6h of onset (PT#_06h): Note: 6h expression is marked by black outline.

The ANOVA analysis of the raw data revealed statistically significant differential expression of 11 microRNAs (miR 210–3p, miR 7–5p, miR 122–5p, miR 140–5p, miR 378i, miR-320e, miR 448, miR 1258, miR 26a-5p, miR 28–5p, miR 510–3p ) across various time points and healthy volunteer comparisons. Recognizing the clinical importance of the hyperacute period within 6 hours from stroke onset, our study concentrated on identifying specific microRNAs that exhibit unique expression profiles during this initial phase, in contrast to subsequent time points and healthy volunteers. Notably, three microRNAs (figure 1) exhibited significant differential expressions within the first 6 hours compared to both the healthy volunteers and other time points.

**Figure 2:**
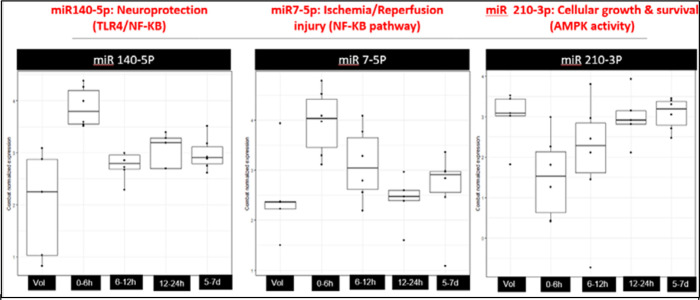
Box plots of miRs 140–5p, 7–5p, and 210–3p showing differential expression at 6h (2nd box plot) compared to healthy controls (1st box) and remaining time points (3rd, 4th and 5th box plots)

Particularly intriguing was the observation that microRNA 140–5p displayed noticeable elevation within 6 hours, gradually normalizing between 12–24 hours and ultimately approaching the volunteer level after 7 days (figure 2). Similarly, microRNA 7–5p exhibited clear overexpression within the first 6 hours, followed by a gradual downtrend towards the volunteer level by 24 hours, maintaining stable expression within a similar range at 7 days. In contrast, microRNA 210–3p demonstrated under-expression at 6 hours, gradually increasing towards the volunteer level over the next 12–24 hours and maintaining that level through 7 days.

### Review of Current Knowledge on miR-140–5p, miR-210–3p, and miR-7–5p in Acute Ischemic Stroke

A concise review of the existing literature regarding the role of these three miRNAs in AIS is presented in Table 2. In animal models simulating ischemic stroke, a significant decrease in miR-140–5p expression was observed within the ischemic core (27–29). Conversely, when examining human serum samples, all studies consistently reported an elevation in serum miR-140–5p levels after cerebral ischemia (30–32). This alignment with our findings suggests the potential utility of miR-140–5p as a diagnostic marker, a notion supported by these consistent outcomes. The observed elevation of miR-140–5p in circulation and its concurrent reduction within the ischemic brain tissue during the initial 6-hour window may reflect a response to ischemic insult. Ischemia/reperfusion injury might trigger the translocation of miR-140–5p from the affected neurons to the circulatory system. This process could be related to the mobilization of inflammatory mediators and growth factors, crucial for the brain’s intrinsic response to ischemic damage.

Moreover, studies have also explored the therapeutic potential of miR-140–5p. Wang et al. demonstrated that administration of encapsulated miR-140–5p could alleviate neuronal damage in subarachnoid hemorrhage(27). Liang et al.’s work showcased that overexpressing miR-140–5p using adeno-associated viruses reduced inflammatory and vascular growth factors in the ischemic mouse hippocampus, inhibiting neurogenesis and capillary density(30). Similarly, Sun et al. revealed that miR-140–5p hinders angiogenesis after cerebral ischemia, potentially contributing to the mitigation of hemorrhagic transformation and edema(28). Additionally, Song et al. provided evidence that miR-140–5p overexpression inhibited neuron apoptosis and decelerated stroke progression(29). While these animal model and in vitro studies show promise for the therapeutic role of miR-140–5p, it is important to note that the limited number of studies and inconsistencies in miR-140–5p delivery methods preclude any definitive conclusions.

As illustrated in Table 2, our literature search found two human-based studies regarding the role of miR-7–5P as a biomarker in stroke. In contrast to our study, Ni et al. observed a reduction in miR-7–5p levels following stroke. However, in contrast to our study, they did not detail the precise timing of sample collection, referring instead to a broader 48-hour window(33). Meanwhile, Chen et al. demonstrated that in humans with intracerebral hemorrhage (ICH), the serum levels of miR-7–5p were significantly higher on day one compared to day 7, demonstrating time dependent evolution in ICH (34). Most studies in animal models of cerebral ischemia and intracerebral hemorrhage have indicated a decrease in miR-7–5P levels in brain tissue samples (33–36). Similarly, in a model of carotid artery injury, miR-7–5p was found to be downregulated when examining carotid endarterectomy samples(37). Similar to 140–5p, the decreases in tissue miR-7–5p levels might be attributed to the release of miR-7–5P from injured tissue into the serum. However, Zhao et al. observed a contrasting trend, with miR-7–5P significantly upregulated in ischemic brain tissue in a time-dependent manner (38). Dharap et al. noted no initial change in miR-7–5P levels, followed by a decline after 12 hours in a rat model of focal ischemia (39). Given these conflicting results in varied models with varied tissue types and sampling time points, the utility of circulating miR-7–5P as a diagnostic biomarker remains uncertain and needs further evaluation.

Several investigations have focused on the therapeutic implications of miR-7–5p. Chen et al. found that miR-7–5p levels were raised by Butylphthalide via intracerebroventricular administration, which contributed to the alleviation of brain edema(34). Xu et al. reported that curcumin regulates miR-7–5p, conferring neuroprotection and ameliorating cognitive deficits in ischemic reperfusion injury. (35). Kim et al. observed that preischemic administration of miR-7 mimics enhanced motor function and diminished lesion volume in young male rats, while post ischemic treatment was effective in reducing brain damage across all rats, improving cognitive outcomes and expediting motor recovery (36).Additionally, Ni et al. demonstrated that elevating let-7c-5p levels via intra-cerebrovascular injection reduced infarct size and lessened neurological impairments (33). Conversely, Zhao et al., studying a rat model of ischemia reperfusion, identified that an increase in miR-7–5p was associated with heightened inflammation, apoptosis, and the exacerbation of ischemic damage(38). Overall, the current body of research on miR 7–5p also reveals variations in miRNA expression profile possibly linked to varied type of biological specimen, disease severity, sampling timepoint, and miRNA profiling techniques.

MiR-210 has also received considerable attention in stroke research, as detailed in Table 2, with investigations encompassing in vitro analyses, animal models, and clinical studies to evaluate its diagnostic, prognostic, and therapeutic potential. Across these studies, a recurrent finding is the elevation of miR-210 expression within brain tissue following cerebral ischemia, including ischemic stroke and hypoxic-ischemic encephalopathy (40–46). In contrast, circulating levels of miR-210 in ischemic stroke patients appear to be suppressed when compared to those of healthy controls (41,42,47,48). This same trend is observed in patients with symptomatic carotid stenosis, where miR-210 is downregulated in carotid fibrous cap tissue (48). This finding underscores the potential of miR-210 as a reliable biomarker for cerebral ischemia. Supporting its diagnostic role, Rahmati et al. established a threshold for miR-210 with a fold change of 0.26, correlating with a modest diagnostic performance characterized by an area under the receiver operating characteristic curve (AUC) of 0.61 and exhibiting 59.62% sensitivity and 65.38% specificity (49). Zeng et al. identified a higher sensitivity at a diagnostic cutoff point of 0.505 for miR-210, achieving 88.3% sensitivity(42). Complementing these studies, Tian et al. confirmed the high diagnostic accuracy of miR-210 for acute cerebral infarction, presenting an AUC of 0.836 (47). These findings collectively point towards the potential of miR-210 as an informative biomarker for the identification of acute ischemic events.

The role of miR-210 in prognostication for ischemic stroke patients has been substantiated by multiple studies. For instance, Rahmati et al. found a positive correlation between elevated miR-210 levels at three months post-stroke and enhanced survival rates.(49). Zeng et al. reported that patients with favorable recovery showed higher miR-210 expression than those with adverse outcomes (42). On the contrary, Tian et al. reported that patients with lower miR-210 expression levels had increased one-year mortality, with miR-210 levels emerging as a robust predictor of mortality (AUC = 0.786) (47). While the current body of research presents variability, likely attributable to insufficient control of confounding variables across different studies, the aggregated evidence nonetheless points to miR-210 as a potentially valuable marker for predicting neurological outcomes in acute ischemic stroke scenarios.

In terms of therapeutic implications, miR-210 has shown potential in both in vitro and animal models as summarized in Table 2. Research by Eken et al. demonstrated the prophylactic effect of miR-210 mimics on carotid plaque stability, suggesting a preventative role against ischemic stroke (48). Pfeiffer et al.’s subgroup analysis revealed that pretreatment with a miR-210–3p mimic substantially mitigated hemispheric swelling and infarct size(40). Similarly, Huang et al. validated the protective effects of miR-210, noting that both pre- and post-treatment with a miR-210 locked nucleic acid (LNA) conjugate led to reduced cerebral infarct and edema, alongside behavioral improvements in mice models of middle cerebral artery occlusion (MCAO)(46). Additionally, Zeng et al. illustrated the efficacy of miR-210 gene transfer in enhancing recovery in transient MCAO models (41). Additionally, research by Li et al. and Zhang et al. has highlighted miR-210’s role in attenuating inflammation and reducing ischemic damage in both in vitro settings and cerebral ischemia models(44,45). Ma et al. demonstrated the neuroprotective effects of exogenous miR-210 mimics in a model of neonatal hypoxic-ischemic brain injury.(43), while Lu et al. documented enhanced function of endothelial progenitor cells under hypoxic conditions when treated with miR-210(50). Yerrapragada et al. further corroborated the neuroprotective role of miR-210 in a hypoxia and reoxygenation model, indicating its therapeutic potential in mitigating hypoxic-ischemic neuronal damage(51). Extending beyond cerebral models, Ujigo et al. found that intracranial administration of miR-210 contributed to functional recovery in cases of traumatic spinal cord injury(52). These studies, underscore the promising therapeutic avenues miR-210 may offer for ischemic stroke intervention.

## Discussion

Our study revealed that, upon comparing each time point against the remaining three time points in acLVO patients and a single time point in healthy volunteers, a total of 11 microRNAs exhibited significantly altered expression across these comparative analyses. Notably, within the first 6 hours of acLVO stroke onset, three microRNAs (140–5p, 7–5p, and 210–3p) exhibited significant differential expression compared to healthy volunteers and other time points. MiRNA 140–5p showed relative increase within the first 6 hours, gradually normalizing between 12–24 hours and reaching volunteer levels within seven days. Similarly, miRNA 7–5p displayed significant overexpression within the first 6 hours, followed by a gradual decline towards volunteer levels by 24 hours, maintaining stable expression within a similar range around seven days. In contrast, miRNA 210–3p demonstrated relative under-expression at 6 hours, gradually increasing towards the volunteer level over the next 12–24 hours and maintaining that level through 7 days.

Our study and existing literature have highlighted the significant roles played by microRNAs (140–5p, 7–5p, and 210–3p) in stroke pathophysiology and therapy. Of note, these microRNAs employ diverse mechanisms to exert their effects. We have provided an overview of the various pathways they operate within stroke and related disorders in Table 2. Many of these pathways are closely linked to inflammation, oxidative stress, cell death, and angiogenesis. Utilizing molecular drug discovery to target these pathways or the microRNAs themselves holds promise as an effective strategy for stroke prevention and treatment. In our study, we have also identified other microRNAs, such as miR 210–3p, miR 122–5p, miR 378i, miR-320e, miR 448, miR 1258, miR 26a-5p, miR 28–5p, and miR 510–3p in association with ischemic stroke. The functions and potential pathways of these miRNAs and other relevant miRNAs are summarized in supplementary file 1. These microRNAs are subjects of ongoing research, aiming to elucidate their roles and mechanisms further.

Our study has several strengths. In this pilot project, we endeavored to meticulously assemble a homogenous cohort of patients, each presenting with anterior circulation acLVO, to maintain uniformity in the stroke phenotype for our analyses. Recognizing the potential for variability introduced by timing, we strictly limited the collection of blood samples to within a 6-hour window following the onset of symptoms, which we hoped would reduce confounding factors related to timing ambiguities. We adopted a longitudinal design for the study, which permitted us to cautiously interpret the evolution of miRNA profiles over time, treating each time point as an intrinsic control against the baseline hyperacute samples. This careful approach, while preliminary, was expected to offer valuable insights into the dynamic changes of miRNAs in this context.

While the study presents intriguing outcomes, it is important to recognize its limitations. A key limitation is the modest cohort size, comprising a total of 29 samples, which may limit the generalizability of the findings. The LVO group included a total of 4 female samples only, which hindered the assessment of potential sex-related differences in miRNA profiles. Although stroke typically occurs in older individuals, the majority of our study’s participants were middle-aged, with one patient being a minor. This distribution may not accurately reflect the age-related risk of stroke in the general population. Additionally, the use of healthy controls who were not matched for stroke risk factors could introduce confounders into the miRNA expression profiles. As Toor et al. indicated, miR-140–5p levels were found to be elevated in stroke patients with diabetes relative to non-diabetic patients (32), suggesting that miRNA expression may differ with underlying risk factors. Moreover, our research was confined to the study of EV encapsulated miRNA and the potential role of non-vesicular, free circulating miRNAs was not investigated, which constitutes an area for further research.

Our literature review disclosed considerable heterogeneity within the corpus of research investigating the role of miRNAs in ischemic stroke. This variation is likely due to a lack of standardization across several critical aspects of study design and methodology. These aspects include the criteria for control group selection, the source of the miRNAs (serum, plasma, CSF or brain tissue), the protocols used for miRNA isolation, the timing of sample collection (ranging from hyperacute to delayed phases), the selection of reference standards (internal and external controls), the choice of detection and quantification techniques (such as Nanostring, Next-Generation Sequencing, or RT-qPCR), and the breadth of the infarct sizes. Additionally, the biological origin of the miRNAs—whether cellular, vesicular, or cell-free—also contributes to the variability of the results, further complicating the interpretation and comparison of findings across studies.

To enhance the reliability of biomarker studies, future investigations should aim for rigorously matched control groups that align with stroke patients’ symptoms and risk factors, utilizing consistent and validated methodologies within a well-defined stroke cohort. Adopting a multi-center design would improve the robustness and applicability of miRNA biomarkers for diagnostic purposes. Additionally, it is crucial to assess the prognostic value of these miRNAs by examining their associations with both radiological findings and clinical outcomes. Implementing miRNA profiles in the elucidation of disease pathways could inform treatment strategies and support timely consultations with patients and their families. Moreover, in-depth mechanistic research is needed to decipher the roles of specific miRNAs in the pathogenesis of acute large vessel occlusion (acLVO) strokes, potentially uncovering novel therapeutic avenues. These efforts will deepen our understanding of miRNA-mediated regulation in stroke and could lead to significant advances in patient care

## Conclusion

In conclusion, our investigation has shed light on the intricate role of miRNAs in stroke pathophysiology, highlighting their potential as biomarkers for acute cerebrovascular events. By identifying 11 microRNAs, particularly miR-140–5p, miR-7–5p, and miR-210–3p, with significant differential expression within 6 hours of stroke onset, our study suggests these miRNAs could potentially serve as valuable indicators for diagnosis and possible targets for therapy, given their involvement in critical pathways like inflammation, oxidative stress, and angiogenesis. Despite promising indications for early detection and stroke management, the limitations of our study call for extensive validation through larger, risk-matched cohorts in multi-center trials. Such rigorous research is essential for confirming miRNAs’ utility as reliable clinical biomarkers and for potentially uncovering new therapeutic strategies that could significantly improve patient outcomes. It is anticipated that the present findings will encourage further detailed exploration of miRNA functions post-ischemic stroke, fostering advancements in clinical approaches and patient care.

## Figures and Tables

**Table 1:** Demographics and clinical characteristics of acLVO patients

Variables/ Patient ID	PT1	PT2	PT3	PT4	PT5	PT6
Age	85	57	45	56	43	14
Gender	Male	Female	Male	Male	Male	Male
Race	White	White	White	White	White	White
IPA (Yes/No)	Yes	Yes	Yes	Yes	Yes	Yes
Biosampling time (pre, intra & post ET)	pre (TICI3)	pre (NT)	post (TICI2b)	pre (TICI2b)	pre (NT)	intra (TICI2b)
Etiology	Cryptogenic	Cryptogenic	Cryptogenic	Cardioembolic	LAA	LAA
HTN	Yes	Yes	No	Yes	No	No
HLD	Yes	Yes	No	Yes	No	No
DM	No	No	No	No	No	No
Atrial fib/Flutter	No	No	No	Yes	No	No
IV Drug use	No	No	No	No	No	No
Smoking	No	Yes	No	No	No	No
Hx of CAD	No	No	No	No	No	No
Anticoagulants	No	No	Yes	No	No	No
Antiplatelets	Yes	Yes	No	Yes	Yes	Yes
Statin Use	Yes	Yes	No	Yes	Yes	Yes
Site of Occlusion	R-ICA	R-MCA	L-MCA	R-MCA	L-ICA	L-MCA
NIHSS at presentation	17	3	17	10	4	1
NIHSS at discharge	8	10	10	10	8	3
MRS at discharge	4	3	4	2	1	2
HT	Yes	No	No	No	No	No

Hypertension (HTN), Hyperlipidemia (HLD), Diabetes Mellitus (DM), Atrial fibrillation/Atrial flutter (Atrial fib/flutter), NIH stroke scale (NIHSS), Modified Rankin Scale (MRS), Hemorrhagic Transformation (HT); Patient (PT)

**Table 2:** Essential methodological aspects and insights from stroke literature investigating the function of three principal miRNAs identified in our study